# Supplementary material for: Vemurafenib and Dabrafenib Downregulates RIPK4 Level
Source: Cancers (Basel). 2023 Feb 1;15(3):918. doi: 10.3390/cancers15030918 (PMC9913565; doi:10.3390/cancers15030918)
Supplement: Supplementary file 1 [file cancers-15-00918-s001.zip › cancers-2181849-supplementary.pdf]

cancers

MDPI

Supplementary material

Vemurafenib and dabrafenib downregulates RIPK4 level

Ewelina Madej [1], Anna A. Brożyna [2], Agnieszka Adamczyk [3], Norbert Wroński [1], Agnieszka Harazin-Lechowska [3], Anna Muzyk [1], Krzysztof Makuch [4], Michał Markiewicz [4], Janusz Rys [3], Agnieszka Wolnicka-Głubisz [1,*]

[1] Department of Biophysics and Cancer Biology, Faculty of Biochemistry, Biophysics and Biotechnology, Jagiellonian University, Kraków, Poland
[2] Nicolaus Copernicus University, Department of Human Biology Torun, Poland
[3] Department of Tumour Pathology, Maria Sklodowska-Curie National Research Institute of Oncology, Krakow Branch, Krakow, Poland
[4] Department of Computational Biophysics and Bioinformatics, Faculty of Biochemistry, Biophysics and Biotechnology, Jagiellonian University, Kraków, Poland

* Correspondence: a.wolnicka-glubisz@uj.edu.pl; Tel.: +48-12-664-65-26; Fax: +48-12-664-69

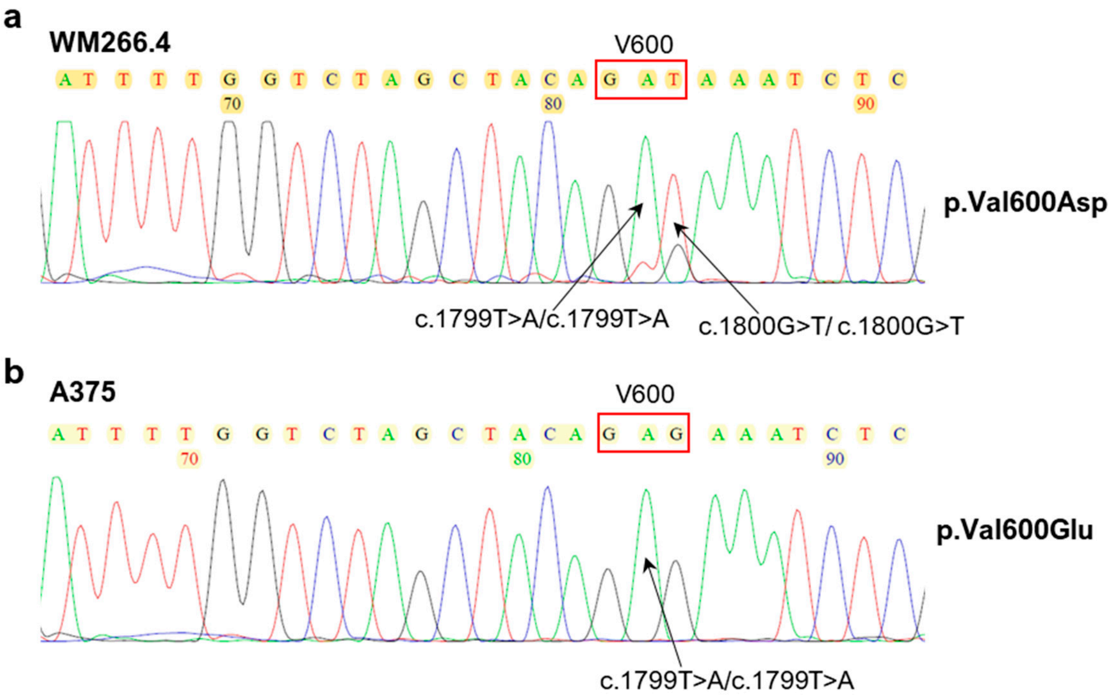

Supplementary Figure S1. Identification of the mutated variant of the BRAF gene. The presence of mutations in the BRAF gene was assessed by Sanger sequencing in melanoma cell line WM266.4 and A375. The codon encoding the amino acid at position 600 of the protein was marked with a red square. The result is presented as a chromatogram of DNA.

Score	Expect	Method	Identities	Positives	Gaps
1406 bits(3640)	0.0	Compositional matrix adjust.	711/786(90%)	744/786(94%)	2/786(0%)

```
Query  1    MEGEGRGRWALGLLRTFDAGEFAGWEKVGSGGFGQVYKVRHVHWKTWLAIKCSPSLHVDD  60
            MEG+G   WAL LLRTFDAGEF GWEKVGSGGFGQVYKVRHVHWKTWLAIKCSPSLHVDD
Sbjct  1    MEGDGGTPWALALLRTFDAGEFTGWEKVGSGGFGQVYKVRHVHWKTWLAIKCSPSLHVDD  60

Query  61   RERMELLEEAKKMEMAKFRYILPVYGICQEPVGLVMEYMETGSLEKLLASEPLPWDLRFR  120
            RERMELLEEAKKMEMAKFRYILPVYGIC+EPVGLVMEYMETGSLEKLLASEPLPWDLRFR
Sbjct  61   RERMELLEEAKKMEMAKFRYILPVYGICREPVGLVMEYMETGSLEKLLASEPLPWDLRFR  120

Query  121  IVHETAVGMNFLHCMSPPLLHLDLKPANILLDAHYHVKISDFGLAKCNGMSHSHDLSMDG  180
            I+HETAVGMNFLHCM+PPLLHLDLKPANILLDAHYHVKISDFGLAKCNG+SHSHDLSMDG
Sbjct  121  IIHETAVGMNFLHCMAPPLLHLDLKPANILLDAHYHVKISDFGLAKCNGLSHSHDLSMDG  180

Query  181  LFGTIAYLPPERIREKSRLFDTKHDVYSFAIVIWGVLTQKKPFADEKNILHIMMKVVKGH  240
            LFGTIAYLPPERIREKSRLFDTKHDVYSFAIVIWGVLTQKKPFADEKNILHIM+KVVKGH
Sbjct  181  LFGTIAYLPPERIREKSRLFDTKHDVYSFAIVIWGVLTQKKPFADEKNILHIMVKVVKGH  240

Query  241  RPELPPICRPRPRACASLIGLMQRCWHADPQVRPTFQEITSETEDLCEKPDEEVKDLAHE  300
            RPELPP+CR RPRAC+ LI LMQRCW  DP+VRPTFQEITSETEDLCEKPD+EVK+ AH+
Sbjct  241  RPELPPVCRARPRACSHLIRLMQRCWQGDPRVRPTFQEITSETEDLCEKPDDEVKETAHD  300

Query  301  PGEKSSLESKSEARPESSRLKRASAPPFDNDCSLSELLSQLDSGISQTLEGPEELSRSSS  360
               KS  E +SE  P   +RLKRASAP FDND SLSELLSQLDSG+SQ +EGPEELSRSSS
Sbjct  301  LDVKSPPEPRSEVVP--ARLKRASAPTFDNDYSLSELLSQLDSGVSQAVEGPEELSRSSS  358

Query  361  ECKLPSSSSGKRLSGVSSVDSAFSSRGSLSLSFEREASTGDLGPTDIQKKKLVDAIISGD  420
            E KLPSS SGKRLSGVSSVDSAFSSRGSLSLSFERE ST DLG TD+QKKKLVDAI+SGD
Sbjct  359  ESKLPSSGSGKRLSGVSSVDSAFSSRGSLSLSFEREPSTSDLGTTDVQKKKLVDAIVSGD  418

Query  421  TSRLMKILQPQDVDLVLDSSASLLHLAVEAGQEECVKWLLLNNANPNLTNRKGSTPLHMA  480
            TS+LMKILQPQDVDL LDS ASLLHLAVEAGQEEC KWLLLNNANPNL+NR+GSTPLHMA
Sbjct  419  TSKLMKILQPQDVDLALDSGASLLHLAVEAGQEECAKWLLLNNANPNLSNRRGSTPLHMA  478

Query  481  VERKGRGIVELLLARKTSVNAKDEDQWTALHFAAQNGDEASTRLLLEKNASVNEVDFEGR  540
            VER+ RG+VELLLARK SVNAKDEDQWTALHFAAQNGDE+STRLLLEKNASVNEVDFEGR
Sbjct  479  VERRVRGVVELLLARKISVNAKDEDQWTALHFAAQNGDESSTRLLLEKNASVNEVDFEGR  538

Query  541  TPMHVACQHGQENIVRTLLRRGVDVGLQGKDAWLPLHYAAWQGHLPIVKLLAKQPGVSVN  600
            TPMHVACQHGQENIVR LLRRGVDV LQGKDAWLPLHYAAWQGHLPIVKLLAKQPGVSVN
Sbjct  539  TPMHVACQHGQENIVRILLRRGVDVSLQGKDAWLPLHYAAWQGHLPIVKLLAKQPGVSVN  598

Query  601  AQTLDGRTPLHLAAQRGHYRVARILIDLCSDVNICSLQAQTPLHVAAETGHTSTARLLLH  660
            AQTLDGRTPLHLAAQRGHYRVARILIDLCSDVN+CSL AQTPLHVAAETGHTSTARLLLH
Sbjct  599  AQTLDGRTPLHLAAQRGHYRVARILIDLCSDVNVCSLLAQTPLHVAAETGHTSTARLLLH  658

Query  661  RGAGKEALTSEGYTALHLAAQNGHLATVKLLIEEKADVMARGPLNQTALHLAAARGHSEV  720
            RGAGKEA+TS+GYTALHLAA+NGHLATVKLL+EEKADV+ARGPLNQTALHLAAA GHSEV
Sbjct  659  RGAGKEAVTSDGYTALHLAARNGHLATVKLLVEEKADVLARGPLNQTALHLAAAHGHSEV  718

Query  721  VEELVSADLIDLSDEQGLSALHLAAQGRHSQTVETLLKHGAHINLQSLKFQGGQSSAATL  780
            VEELVSAD+IDL DEQGLSALHLAAQGRH+QTVETLL+HGAHINLQSLKFQGG   AATL
Sbjct  719  VEELVSADVIDLFDEQGLSALHLAAQGRHAQTVETLLRHGAHINLQSLKFQGGHGPAATL  778

Query  781  LRRSKT  786
            LRRSKT
Sbjct  779  LRRSKT  784
```

Supplementary Figure S2. Comparison of full-length of amino acid sequences of human RIPK4 (Sbjct) and BRAF (Query) using BLAST.

Tabele S1. Antibodies used for Western Blot and immunofluorescence analyses.

Antibody	Source	Dilution	Cat. No.	Company
anti-RIPK4	rabbit	1:2000	12636	Cell Signaling Technology
anti-phospho-RB1 (Ser807/811)		1:4000	8516	
anti-RB1	mouse		9309	
anti-CDK2	rabbit	1:2000	2546	
anti-phospho-BRAF (Ser445)			2696	
anti-phospho-MEK (Ser217/221)			9154	
anti-phospho-p44/24 (ERK1/2) (Thr202/Tyr204)			9101	
anti-p44/24 (ERK1/2)			9102	
anty-PEBP-1	mouse	1:4000	sc-376925	SantaCruz Biotechnology
anti-phospho-PEBP-1 (Ser 153)			sc-135779	
anti-GAPDH	rabbit	1:2000	5174	Cell Signaling Technology
anti-phospho-FAK (Tyr397)			8556	
anti-phospho-AKT (Ser 473)			4060	
anti-AKT			4685	
HRP-conjugated goat anti-rabbit	goat	1:4000	7074	
HRP-conjugated goat anti-mouse			554002	BP Pharmingen
Alexa 555-conjugated Anti-Ki67	mouse	1:10	558617	BD Biosciences

Tabele S2. List of uses TaqMan probe purchased from Thermo Fisher Scientific / Invitrogen.

Gene	ID TaqMan probe
RIPK4	Hs01062501_m1
CDK2	Hs01548894_m1
CDK2AP1	Hs00934308_m1
CDK14	Hs00953418_m1
CDK6	Hs01026371_m1
RIPK1	Hs01041869_m1
RIPK3	Hs00179132_m1
BCL-2	Hs00608023_m1
BAD	Hs00188930_m1
MCL1	Hs06626047_g1
GAPDH	Cat. No. 4326317E
